# Role of dimethyl fumarate in the treatment of glioblastoma multiforme: A review article

**Published:** 2019-07-06

**Authors:** Reza Ahmadi-Beni, Ali Najafi, Seyed Mehrdad Savar, Niayesh Mohebbi, Alireza Khoshnevisan

**Affiliations:** 1 Department of Medical Genetics, School of Medical Sciences, Tarbiat Modares University, Tehran, Iran; 2 Department of Medical Genetics, School of Medicine, Tehran University of Medical Sciences, Tehran, Iran; 3 Student Scientific Research Center, Tehran University of Medical Sciences, Tehran, Iran; 4 School of Pharmacy, Tehran University of Medical Sciences, Tehran, Iran; 5 Department of Clinical Pharmacy, School of Pharmacy, Tehran University of Medical Sciences, Tehran, Iran; 6 Department of Neurosurgery, Shariati Hospital, Tehran University of Medical Sciences, Tehran, Iran

**Keywords:** Brain Neoplasms, Glioblastoma, Fumarates, Dimethyl Fumarate, Neuroprotective Agents, Drug Repurposing

## Abstract

Glioblastoma multiforme (GBM), the most frequent malignant and aggressive primary brain tumor, is characterized by genetically unstable heterogeneous cells, diffused growth pattern, microvascular proliferation, and resistance to chemotherapy. Extensive investigations are being carried out to identify the molecular origin of resistance to chemo- and radio-therapy in GBM and find novel targets for therapy to improve overall survival rate. Dimethyl fumarate (DMF) has been shown to be a safe drug with limited short and long-term side effects, and fumaric acid esters (FAEs), including DMF, present both anti-oxidative and anti-inflammatory activity in different cell types and tissues. DMF has also anti-tumoral and neuroprotective effects and so it could be repurposed in the treatment of this invasive tumor in the future. Here, we have reviewed DMF pharmacokinetics and different mechanisms by which DMF could have therapeutic effects on GBM.

## Introduction

Glioblastoma multiforme (GBM) establishes more than half of the malignant gliomas, the most malignant and aggressive primary brain tumor, and is mainly a malignancy of older ages.^1^^,^^2^ It is characterized by genetically unstable heterogeneous cells, diffused growth pattern, microvascular proliferation, and resistance to chemotherapy.^3^^,^^4^ Current therapy includes maximal surgical resection followed by chemoradiotherapy, but only less than 5% of patients with GBM survive 5 years postdiagnosis and the median survival is about 15 months.^2^^,^^5^

Intensive investigations are being carried out to identify the molecular origin of resistance to chemo- and radio-therapy in GBM, looking into novel therapeutic targets and also new drugs that may improve overall survival rate.^6^^-^^8^ 

Dimethyl fumarate (DMF) is an anti-inflammatory and safe medication that has been used for many years in the treatment of psoriasis.^9^^,^^10^ Studies reveal that the beneficial effects of this fumaric acid ester (FAE) arise from its potency to reduce the T cell-mediated inflammatory gene expression, including inflammatory cytokines and chemokines. 

**Figure 1:**
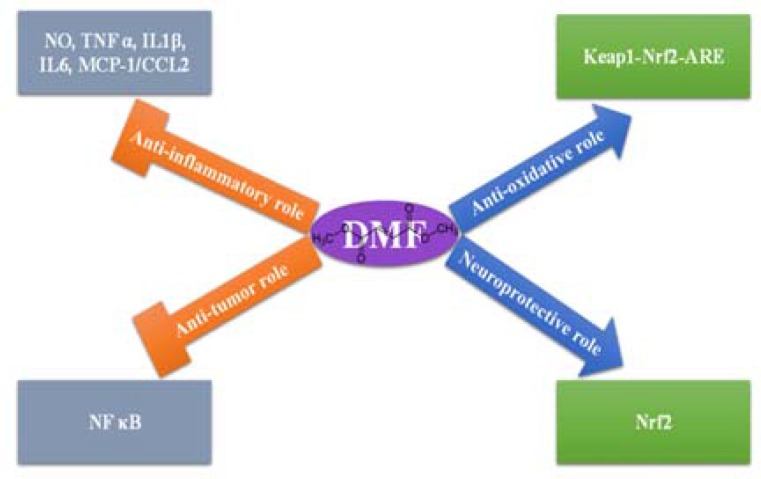
Four main molecular pathways which make dimethyl fumarate (DMF) a suitable anti-cancer drug for glioblastoma multiforme (GBM) therapy

DMF is a drug with an acceptable safety and its short and long-term side effects are few [most commonly consisting of flushing, gastrointestinal (GI) complaints, and leukocytopenia].^11^^-^^13^ Regarding its good safety profile, a formulation of DMF called BG-12 was developed for the treatment of multiple sclerosis (MS),^13^^,^^14^ and then proposed to be repurposed for GBM treatment.^   15 ^ Recently different mechanisms for the role of DMF in neuroprotection and tumor cell destruction have been demonstrated (Figure 1). Here, we review some papers that have been published by authors investigating mechanisms that could be beneficial in the treatment of GBM by DMF, but first we discuss a little about the pharmacokinetics of DMF.

**Pharmacokinetics of DMF**

DMF is available as a delayed-release capsule under the trade name of Tecfidera which is approved by Food and Drug Administration (FDA) to be used for relapsing remitting MS (RRMS) therapy.^        16 ^ DMF can easily enter cells as it is a highly lipophilic substance.^   17 ^ However, once DMF releases from its capsule (as a drug), it undergoes rapid pre-systemic hydrolysis with the help of ubiquitously-found esterases in GI tract, tissues, and blood.^18^^,^^19^ The resulting active metabolite is monomethyl fumarate (MMF). This conversion takes place very fast and as a result, DMF is not detectable in plasma. So, all related pharmacokinetic information is gathered from MMF which is a negatively-charged molecule. MMF cannot cross cell membrane, but it is a hydroxycarboxylic acid receptor 2 (HCA2) agonist and may exert its effects through a HCA2-mediated manner.^        20 ^ Also, MMF has some HCA2-independent effects like activating the pathway of nuclear-factor (erythroid-derived 2)-related factor-2 (Nrf2).^21^^,^^22^ 

Following oral administration of Tecfidera, a median time of 2 to 2.5 hours is needed to peak plasma concentration (T_max_). In case of Tecfidera, the peak plasma concentration (C_max_) is decreased with high-fat high-calorie meal by 40% and the T_max_ is delayed from 2.0 hours to 5.5 hours. During fed state, the flushing occurrence could be diminished by about 25%.^19^^,^^23^ 

MMF is further metabolized through the citric acid cycle (Krebs cycle). MMF, together with fumaric, glucose, and citric acid are of the most metabolites in plasma. The primary path of removal is by exhalation of carbon dioxide (CO_2_) that accounts for about 60% of Tecfidera dose, and the other routes include renal and fecal elimination.^   23 ^

**GBM heterogeneity**

Tumor heterogeneity poses one of the most important obstacles in the definitive diagnosis and targeted treatment of GBM, making differences happen in treatment response and outcome. Understanding and deciphering tumor heterogeneity can bring forth a new patient-tailored treatment regimen.^24^^,^^25^ In addition to intertumoral heterogeneity which refers to the difference in mutation profiles between patients with the same tumor type, allowing molecular subtype classification, there is also intratumoral heterogeneity referring to the difference in genetic alterations between cancer cells within a tumor.^24^^,^^26^ Intratumoral heterogeneity changes during usage of therapy which presents challenges in the setting of GBM recurrence, as recurrent tumor cells usually exhibit resistance to the traditional treatment options and express different mutations in comparison with the original tumor.^24^^,^^27^ This intratumoral heterogeneity has wide significance for the clinical practice outcome of patients, particularly in the current personalized medicine perspective according to analysis of genomic material of an individual cancer biopsy.^        28 ^

Glutathione S-transferases (GSTs) [GSTs are detoxification agents that are used as catalyzers to conjugate the reduced form of glutathione (GSH) to xenobiotic substrates] which are induced by DMF, were investigated in psoriasis and it has been observed that Val/Val GSTP1 polymorphism is associated with non-responsiveness to treatment with this FAEs.^   17 ^ This polymorphism has also been suggested as a potential biomarker predicting the status of responsiveness to DMF in other diseases.^   17 ^ There is no information about the impact of GBM heterogeneity on response to DMF therapy and comprehensive studies on this field are required.

**Anti-tumor role of DMF**

In addition to its effects on the adaptive immune response, DMF also represses the inherent inflammatory responses of glial cells via decreasing the nuclear factor kappa B (NF-κΒ) signaling pathways activation.^29^^,^^30^ The DMF action is conducted through suppression of signals which are transmitted by the Rel proteins, and also by suppressing the complex of inhibitor of κΒ (IκΒ) kinase (IKK) and translocation of NF-κΒ to the nucleus, that results in halting gene transcription. NF-κΒ complexes are normally situated inside the cell cytoplasm by the inhibitory IκΒ complex. Inflammatory mediators cause phosphorylation of IκΒ complex through IKK action, resulting in ubiquitin-mediated degradation of IκΒ. This allows the NF-κΒ to translocate into the nucleus and subsequently leads to the downstream transcription activation.^        31 ^ DMF decreases inflammatory mediators such as tumor necrosis factor-α (TNF-α), interleukin-1β (IL-1β), nitric oxide (NO), and IL-6, and via suppression of NF-κΒ, it can induce apoptosis in several types of cell like T cells.^   32 ^ DMF has been shown to limit the in vitro and in vivo growth of melanoma.^        33 ^ Correlation of NF-κΒ activity with angiogenesis, cell proliferation, and anti-apoptosis in different solid tumors has been demonstrated by previous studies.^31^^,^^34^^,^^35^ The ability to suppress Rel protein pathway reduces NF-κΒ nuclear translocation, and transcription inhibition of inflammatory cytokines is possible antitumor mechanism of DMF.^36^^,^^37^ Inhibiting angiogenesis via suppressing NF-κΒ in endothelial cells may also halt tumor progression.^38^^,^^39^ Suppression of NF-κΒ in endothelial cells reduces expression of vascular endothelial growth factor receptor-2 (VEGFR-2), inhibits migration of endothelial cells, and stimulates endothelial cell apoptosis, which may elucidate why DMF may be valuable in proliferative diseases including psoriasis, rheumatoid arthritis (RA), tumor growth, and metastasis.^40^^,^^41^ 

There are a few studies investigating the effects of DMF in gliomas which have shown that cytotoxicity of other antitumor agents is increased by pretreatment with DMF.^   38 ^ Ghods et al.^42^ in a study analyzed the potential antitumor properties of DMF in several glioma models by evaluating its effects on proliferation, cell lysis, and differentiation. Human glioblastoma A172, mouse glioma Gl261, and patients’ glioblastoma cells were exposed to DMF at therapeutic (100 μm) and supra-therapeutic concentrations (300 μm) for evaluation of proliferation, cellular death, and differentiation. The lactate dehydrogenase (LDH) cell lysis assay and 5-bromo-2’-deoxyuridine (BrdU) proliferation assay were also used. Differentiation was evaluated by immunocytochemistry (ICC). In the study, proliferation considerably decreased and tumor cell death significantly increased in all tumor cell lines after exposure to DMF. A significant decrease in CD133 expression was observed in addition to a decline in the expression of NF-κΒ after exposure to DMF. Also, a drastic impact of DMF on decreasing the formation of both neurospheres and the expression of CD133 cells in human glioblastoma cells was demonstrated. Results revealed that DMF might suppress tumor proliferation by diminishing these CD133-marked glioblastoma stem cells (GSCs) in the tumor.^   42 ^ These findings warrant further studies on the DMF effects on several hallmarks of GSCs in more detail as new approaches to isolate and characterize tumoral cells that satisfy the functional characteristics of GSCs continue to be explored.^43^^,^^44^ 

In another study in four glioblastoma cell lines, cytotoxicity of the bioreductive antitumor agents, mitomycin C (MMC) and streptonigrin (SN), in presence or absence of DMF as inducer of DT-diaphorase (DTD) (i.e., an obligate two-electron reductase that bioactivates chemotherapeutic quinines) was compared with the 1,3-bis(2-chloroethyl)-1-nitrosourea (BCNU) as a usual chemotherapeutic agent. A significant increase in the cytotoxicity of SN and MMC was observed in glioblastoma cell lines when using DMF in the pretreatment stage, but there was no effect on the BCNU cytotoxicity. In three, out of four, studied glioblastoma cell lines, spectrophotometric measurements showed that DMF drastically increased cytochrome b5 reductase (CYB5R) and DTD activity and reduced GST.^   45 ^ When DTD inhibitor, dicumarol, is added, it considerably suppresses cytotoxic effects of SN and MMC, and also reverses the increased cytotoxicity of either combination of DMF with SN or MMC in the studied glioblastoma-derived cell lines. Combination of CYB5R and DTD inducers with bioreductive antitumor agents could be a promising strategy for the treatment of GBM. MMC does not penetrate an intact blood-brain barrier (BBB) and so, is not known to be an active agent against glioblastoma. SN can traverse the BBB, but because of its myelotoxicity cannot be used clinically without limitation. Temozolomide is a clinically-developed alkylating agent. This prodrug is the analog of imidazotetrazine that in solution spontaneously produces a triazine derivative [5-(3-methyltriazen-1-yl)-imidazole-4-carboxamide (MTIC)]. It goes across BBB with concentrations in the central nervous system (CNS) near 30% of plasma concentrations.^   46 ^ Then, it renders monomethylation of deoxyribonucleic acid (DNA) purine bases (N3-adenine, O6-guanine, N7-guanine).^        47 ^ MMF enhances the toxicity of temozolomide and ionizing radiation.^   15 ^ Another drug that has been experimentally used is bevacizumab (Avastin). Bevacizumab directly binds to vascular endothelial growth factor A (VEGFA) to inhibit its angiogenicity. Inherent and acquired resistance to temozolomide and bevacizumab, in addition to different side effects, presents major obstacles to successful treatment.^        26 ^ Nevertheless, for avoiding these obstacles, concomitant use of DMF with SN, temozolomide or bevacizumab, or a combination of them warrants further study, that may also permit administration of lower doses of the chemotherapeutic agents which will decrease systemic toxicity.^   38 ^ The only phase I clinical trial using DMF in patients with GBM showed that it might be safely used in combination with radiation and temozolomide.^   48 ^ Also, recently, research is on the way for enhancing DMF bioavailability and delivery to improve its concentrations in the brain.^        49 ^

These findings suggest that DMF may be considered for further antitumor investigations and it presents a new treatment approach for brain tumors. These studies have some limitations including that they are entirely in vitro investigations. As a result, in vivo evaluation of DMF effects on GBM is needed to be carried out.^   42 ^

**Neuroprotective role of DMF**

DMF due to its neuroprotective role might support neuronal survival after operation in brain tumors including GBM. One of the examples of neuroprotective properties of DMF is in ischemia. DMF protects the BBB integrity and so attenuates cerebral edema formation. At a first glance, improved vascular permeability seems to assist access of erythropoietin (EPO) and some other neuroprotective factors to the brain parenchyma and looks to be favorable for neuronal survival. Nevertheless, edemogenesis causes elevated intracranial pressure (ICP) and unfavorable effects on oxygen distribution, and consequently the initial neuroprotective impact would be abolished. FAEs [methyl hydrogen fumarate (MHF) and DMF] have anti-oxidative and anti-inflammatory activities in different tissues and cell types such as neurons and astrocytes confronted to oxidative stress situations. Moreover, preconditioning with DMF diminishes the formation of the proinflammatory mediators including IL-6, IL-1β, NO, and TNF-α in lipopolysaccharide (LPS)-activated astrocytes and microglia.^        50 ^ Also, MMF puts freshly isolated activated microglia to death and reduces TNF-α, IL-6, and TGF-β production,^   15 ^ and the last one prevents TGF-β-induced ZEB1-dependent mesenchymal transdifferentiation in GBM that causes tumor invasion.^        51 ^ In an in vivo study, an acute ischemic stroke model for BBB breakdown and cerebral edema formation was used. DMF systemic pre-treatment decreased formation of edema throughout ischemic stroke and reduced the activity of brain tissue matrix metalloproteinases (MMPs) upon ischemic stroke.^        52 ^ It also prevented the disruption of inter-endothelial tight junctions (TJs) during ischemic stroke by triggering the antioxidant nuclear-factor Nrf2 pathway. Findings denoted that neuronal apoptosis, elicited by ischemic stroke, was diminished by systemically-applied DMF.^        52 ^ DMF, by motivating the redox-sensitive transcription factor Nrf2, impedes inflammatory cytokines and so, decreases migration of leukocytes. In fact, increased Nrf2 activity is the cause of protection of the BBB. Proinflammatory factors such as monocyte chemoattractant protein-1 (MCP-1/CCL2) and TNF-α in astrocytes and endothelial cells exposed to ischemic condition are abolished by DMF. DMF and its primary metabolite, MMF, improve the in vitro survival of neurons exposed to oxidative stresses including hydrogen peroxide (H_2_O_2_) and glutamate toxicity.^21^^,^^52^ Besides, it has been shown that FAEs do not activate the pathway of hypoxia-inducible factor (HIF); so, it seems that the pathway does not participate to FAE-mediated neuroprotection significantly. ^53^^,^^54^ 

In recent years, studies revealed that systemic DMF therapy successfully improved neurological recovery, decreased brain edema, and improved resolution of hematoma in rodent models of acute intracerebral hemorrhage (ICH). ^53^^,^^55^ These and some other evidence support the idea that DMF helps neuronal survival in apoptotic processes and slowly-progressing diseases. ^21^^,^^52^^,^^56^ 

The inflammatory response and oxidative stress can cause brain damage in subarachnoid hemorrhage (SAH) and DMF also significantly ameliorates the early brain damage and learning deficiencies in animal models of SAH.^        57 ^ This neuroprotective effect is implemented through activation of the Kelch-like ECH-associated protein 1–Nrf2–antioxidant-response element (Keap1-Nrf2-ARE) system, which causes the downregulation of oxidative stress and inflammation. In one study, SAH model in rats was achieved by 300 μl autologous blood injection into their chiasmatic cistern. In the group treated by DMF, after the onset of SAH, rats were given 15 mg/kg DMF through oral gavage twice daily for 2 days. Brain edema, BBB damage, cortical apoptosis, necrosis of the neurons, learning deficiencies, and alterations in the Keap1-Nrf2-ARE pathway were studied. Treatment with DMF after SAH, significantly upregulated the Keap1-Nrf2-ARE signaling-related genes expressions.^        57 ^

Furthermore, antioxidant activity of DMF is also applied through binding of Nrf2 to promoter-located AREs of protection genes including nicotinamide adenine dinucleotide phosphate (NADPH)-quinone-oxidoreductase-1 (NQO-1)^        37 ^ and heme oxygenase-1 (HO-1). It finally increases the intracellular levels of antioxidant GSH.^11^^,^^56^^,^^58^ 

FAE treatment leads to upregulation of the detoxification enzyme NQO-1 and reduction of the neurotoxic agent NO. DMF also upregulates HO-1, as an anti-stress protein in the human peripheral blood mononuclear cells (PBMCs). DMF has an important inhibitory influence on LPS-activated NO burst in microglia and causes apoptosis in PBMCs. ^59^^,^^60^ 

It is reported that FAEs show protective effects in chronic experimental autoimmune encephalomyelitis (EAE). Both DMF and MHF have a noteworthy beneficial influence on the disease course, and histology shows a significantly decreased macrophage inflammation in the spinal cord.^        60 ^

**Side effects**

Generally, DMF is a safe drug with acceptable tolerability profile and its short and long-term side effects are few. 13^,^^23^ Nevertheless, DMF may cause flushing, rash, lymphocytopenia, albumin urine presence, erythema, and GI events such as nausea, abdominal pain, vomiting, dyspepsia, and diarrhea, but the occurrence of adverse effects are not usually much higher than placebo-treated control groups.^13^^,^^19^^,^^61^ The occurrence of GI events in the early course of treatment (primarily in first month) is higher and commonly diminishes through the time in DMF-treated patients, in comparison to placebo. ^62^^,^^63^ The serious GI events incidence was 1% in DMF-treated patients.^   23 ^ It is suggested from a phase IV clinical trial that GI events seen due to DMF could be well managed with common symptomatic therapy.^   64 ^

## Conclusion

Glioblastoma is a highly-malignant and difficult to treat brain tumor, and despite many investigations on its therapy, only a few drugs with little success have been used clinically for its management. Although development of novel drugs is a matter of importance, finding eligible drugs is a matter of time and budget. Considering these difficulties, repurposing FDA-approved drugs might be a remedial shortcut in comparison with newly-developed ones. DMF, as an FDA-approved drug, is ready for clinical research to be considered as a therapeutic and adjuvant option for GBM therapy. DMF is a FAE which regarding its anti-tumoral and neuroprotective effects in GBM, more studies are necessary to clear its molecular functions especially for suppressing NF-κΒ and triggering Nrf2. Finally, like GBM heterogeneity, other challenges of GBM therapy, like bypassing BBB, deserve equal attention to achieve effective drug concentrations in the tumor tissue for fully effective utilization of DMF in GBM eradication setting.

## Notes:

**How to cite this article:** Ahmadi-Beni R, Najafi A, Savar SM, Mohebbi N, Khoshnevisan A. Role of dimethyl fumarate in the treatment of glioblastoma multiforme: A review article. Iran J Neurol 2019; 18(3): 127-33.
